# Deep learning-based method for analyzing the optically trapped sperm rotation

**DOI:** 10.1038/s41598-023-39819-7

**Published:** 2023-08-03

**Authors:** Jiangcheng Zhao, Chuanbiao Bai, Zhiguo Zhang, Qingchuan Zhang

**Affiliations:** 1 CAS Key Laboratory of Mechanical Behavior and Design of Materials, Department of Modern Mechanics, University of Science and Technology of China, Hefei, 230027 China; 2 School of Biomedical Engineering, Anhui Provincial Institute of Translational Medicine, Anhui Medical University, Hefei, 230027 China

**Keywords:** Biomedical engineering, Optical imaging

## Abstract

Optical tweezers exert a strong trapping force on cells, making it crucial to analyze the movement of trapped cells. The rotation of cells plays a significant role in their swimming patterns, such as in sperm cells. We proposed a fast deep-learning-based method that can automatically determine the projection orientation of ellipsoidal-like cells without additional optical design. This method was utilized for analyzing the planar rotation of trapped sperm cells using an optical tweezer, demonstrating its feasibility in extracting the rotation of the cell head. Furthermore, we employed this method to investigate sperm cell activity by examining variations in sperm rotation rates under different conditions, including temperature and laser output power. Our findings provide evidence for the effectiveness of this method and the rotation analysis method developed may have clinical potential for sperm quality evaluation.

## Introduction

Optical tweezers (OT) have been widely researched for trapping and manipulating micro-particles and microorganisms such as polystyrene beads, yeast cell, sperm and Escherichia coli^1–4^. During the reproductive process, sperms need to meet the egg through the female reproductive tract. In this process, sperm with low motility will be screened out by barriers such as cervical mucus^5^. Extensive research has been conducted on the dynamics of sperm cells trapped in optical tweezers, encompassing studies investigating diverse aspects such as chirality and motility force^6,7^. This research direction has potential value in the single-sperm quality examination, which is crucial to the assistant reproductive technologies like intracytoplasmic sperm injection (ICSI)^8^. Currently, the main method for quickly tracking the movement of the head of a sperm cell involves tracking its centroid, which uses traditional clustering algorithms to segment^9,10^. Then Nascimento et al. employed this method to calculate the curvilinear velocity(VCL) of the head to characterize sperm activity^11^. However, In the high numerical aperture objective lens field of view, traditional segmentation methods have poor segmentation results due to complex background noise. Besides, for more complex motion patterns of sperm, such as rotation, the additional optical set-up should be designed, and corresponding algorithms to determine the three-dimensional motion of the sperm usually perform relatively slowly^12^.

Deep learning has been widely applied in the field of medical cell image segmentation, especially for particular cell counting^13^, liver and liver-tumor segmentation^14^, brain and brain-tumor segmentation^15^ etc. And compared with traditional methods, it has superior segmentation performance and robustness^16,17^.This tool can also be combined with optical tweezers and applied to axial localization of microspheres^18^, optical force prediction of the optical tweezers^19^, and trap stiffness measurement^20^. In this study, we propose highly efficient method that extracting the orientation of the sperm head using deep learning-based segmentation. We combine it with optical tweezers to dynamically trap sperms and analyze the rotational motility of individual sperm directly and simultaneously. This method is also suitable with other ellipsoidal-like cells or bacteria like Escherichia coli. Our experiments analyzed the difference in rotational angular speed of sperm cells at different laser output power and verified that our method has potential applications in clinical research for sperm motility quantification and single sperm motility detection.

## OT set-up

**Figure 1:**
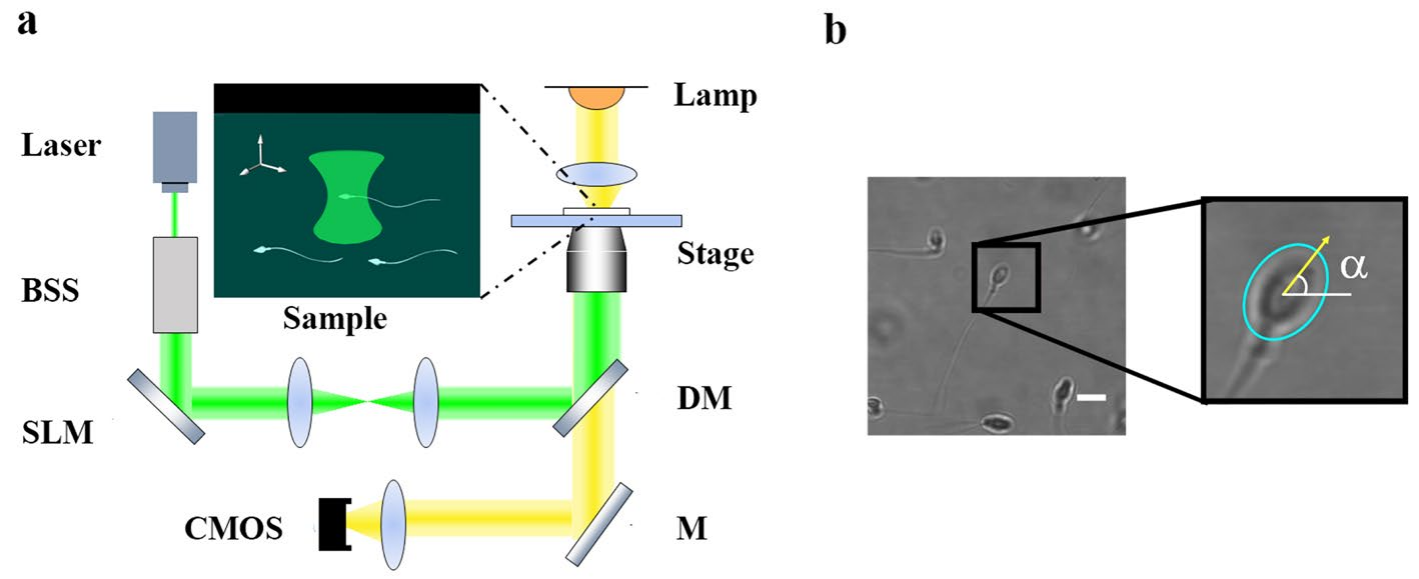
The experimental apparatus (**a**) and the schematic diagram of phase angle extraction (**b**), the white scale bar represents 10 $\upmu$m.

To obtain the projection phase information of the sperm, we used a self-designed optical tweezers system as shown in Fig. 1a. In our experimental setup, we employed a continuous wave laser (Coherent Verdi G, 2W) to generate a 532-nm laser beam. This laser beam was directed into a beam shaping system (BSS) for beam expansion. The resulting linear polarized laser beam was then directed onto a dichroic mirror, which reflected it onto the back aperture of an oil immersion microscope objective (Nikon, 100$\times$ oil immersion; NA = 1.4; WD = 0.13 mm). The beam is modulated through a reflective and pure phase-only liquid crystal spatial light modulator (Holoeye, HED6010-L-VIS) in the shaping system. The sample solution of interest was loaded onto a sample pool and positioned at the back focal plane of the oil mirror, which was controlled using a voltage-controlled stage (Thorlabs; ZFM2030). The illumination of the sample chamber was provided by a LED light source and the imaging of the sample was performed using a CMOS camera (Sentech; STC-mbs241U3V; 163 fps).

## Rotational angular speed measurement using U-Net++

Prior to the experiment, we calibrated the position of the optical trap and used a 150 $\times$ 150 pixel region around the trap center as the region of interest (ROI) to exclude interference from other swimming sperms, as shown in Fig. 1b. For the extracted image area, we used image segmentation to perform contour detection to obtain the projection phase of the head shape. Then, the rotational angular speed was calculated from the phase difference between consecutive frames.

### Segmentation of sperm images using U-Net++

**Figure 2:**
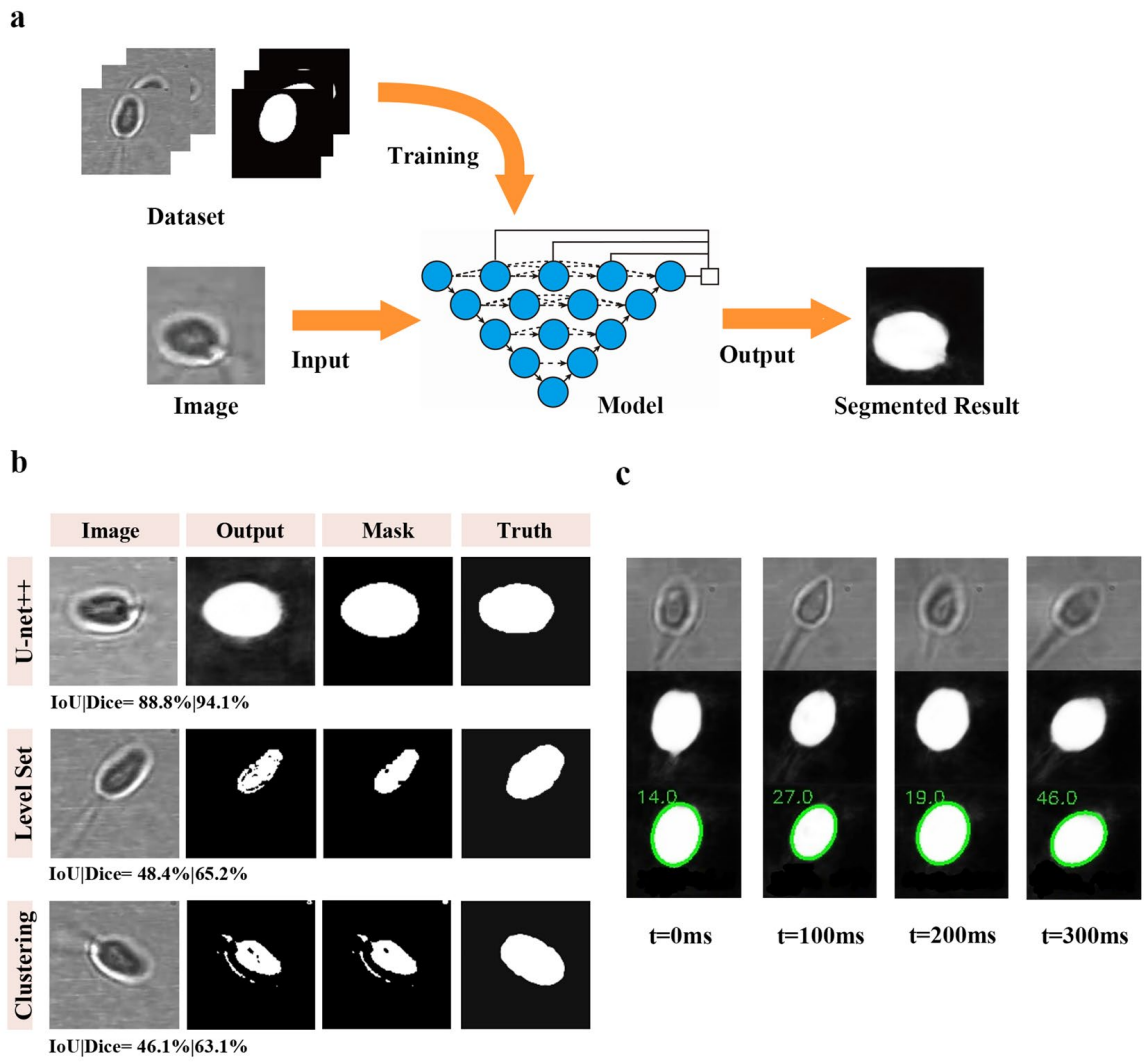
Diagram of the model training process (**a**); showing the training set used for training, including the original image and pre-segmented image, and the image segmentation process below. Qualitative comparison among U-net++, Level set, and Clustering method (**b**); showing segmented result and final mask output for the trapped sperm. The corresponding quantitative scores are provided at the bottom of each prediction (IoU|Dice). Frames of a sperm trapped at 0 ms, 100 ms, 200 ms, and 300 ms (**c**), with the original images shown at the top, the segmented images in the middle, and the contour extracted results at the bottom. The green label represents the phase of the fitted ellipse.

To achieve faster and more accurate segmentation results, we introduced a deep-learning segmentation algorithm. This is because traditional gray threshold segmentation, such as level set and clustering method, is greatly affected by noise in the segmentation of high-magnification cell images under high-NA lens imaging, as shown in Fig. 2b. Moreover, these methods rely on manual or adaptive adjustment of parameters, which may not be effective in complex scenes. Deep learning-based image segmentation algorithms have become a hot topic in recent years. In this study, we used the U-Net++ network for image segmentation. U-Net++ is a supervised learning network based on the fully convolutional neural network, U-Net. By redesigning the skip connections, it aggregates information features of different semantic scales in the decoder sub-network, forming a highly flexible feature fusion solution, and has performed well on many biological datasets and backbone architectures^21^. As a type of supervised learning, the training of this model requires a certain amount of data to generate better segmentation results. Figure 2a shows the schematic diagram of our solution. The loss function we used is the hybrid loss which combined the cross-entropy loss and soft dice-coefficient loss. The hybrid loss is defined as^22^

$$\begin{aligned} L(Y,P)=-\frac{1}{N}\sum \limits _{c=1}^{2}{\sum \limits _{n=1}^{N}{\left( {{y}_{n,c}}\log {{p}_{n,c}}+2\frac{2{{y}_{n,c}}{{p}_{n,c}}}{{{y}_{n,c}}^{2}+{{p}_{n,c}}^{2}}\right) }} \end{aligned}$$

where ${{y}_{n,c}}\in \text {Y}$ and ${{p}_{n,c}}\in \text {P}$ denotes the target labels and predicted probabilities for class c (here just two classes) and *n*th pixel in the training batch. Our training dataset consisted of 125 images of trapped sperm cells with a size of 120 $\times$ 120 pixels and the corresponding manually segmented results. The network depth was set to 4 layers, and the optimal network parameters were calculated using cross-validation. After processing the segmented sperm images, a threshold method can be used for binarization to extract the mask. Despite the small size of the training dataset, the segmentation results are remarkable, particularly when compared to the other two models, as is shown in Fig. 2b, where the intersection over union (IoU) and Dice coefficients can reach around 90%. The IoU and Dice coefficients are defined as follows^23^, where the S represents the segmented mask and T represents the truth mask. For each of them, a higher value indicates a higher degree of overlap and similarity between the segmentation result and the reference mask

$$\begin{aligned}{} & {} IoU(S,T)=\frac{|S\bigcap T|}{|S \bigcup T|},\\{} & {} Dice(S,T)=\frac{2|S\bigcap T|}{|S| + |T|} \end{aligned}$$

The level set and clustering models in the study utilized modules provided by ImageJ 1.53v^24^. This approach can achieve rapid and accurate image segmentation in milliseconds by deploying on high-performance GPUs, achieving real-time segmentation, as shown in Table 1. The experiments are performed using NIVIDA GeForce RTX 2080 GPU with 8 GB memory.

**Table 1:** Comparison of segmentation models based on IoU and cost time.

Model	Average IoU (%)	Average cost time/s
U-net++	88.5	0.023
Level set	48.1	0.071
Clustering	45.7	0.026

### Rotational angular speed determined by elliptical fitting

The projected orientation of the sperm needs to be determined from the segmented image. The human sperm head is flat and elliptical, with a length of about 4–5 $\upmu$m and a width of about 2.5–3.5 $\upmu$m^25^. This elliptical shape is different from the circular shape of typical cells, so the two-dimensional orientation of the sperm can be determined visually. In this study, the elliptical fitting tool provided by OpenCV^26^ is used to approximate the shape of the sperm head in the grayscale image obtained from the segmented sperm image, and the phase information $\alpha$ of the head is obtained, as shown in Fig. 1b. Figure 2c depicts a series of images of segmented sperm at four different time points, along with the results of ellipse fitting. It can be observed that the phase is accurately extracted. Despite the fact that the segmented result appears oval when the sperm is in a sideways position, the phase is still acquired accurately. The absolute angular speed is then obtained using the forward difference method, as shown in the following equation,where k means the *k*th frame, fps denotes the recording rate of 163 frames per second:

$$\begin{aligned} \Omega (k)=|\alpha (k+1)-\alpha (k)|/(2\pi )*fps \end{aligned}$$

## Result and discussion

**Figure 3:**
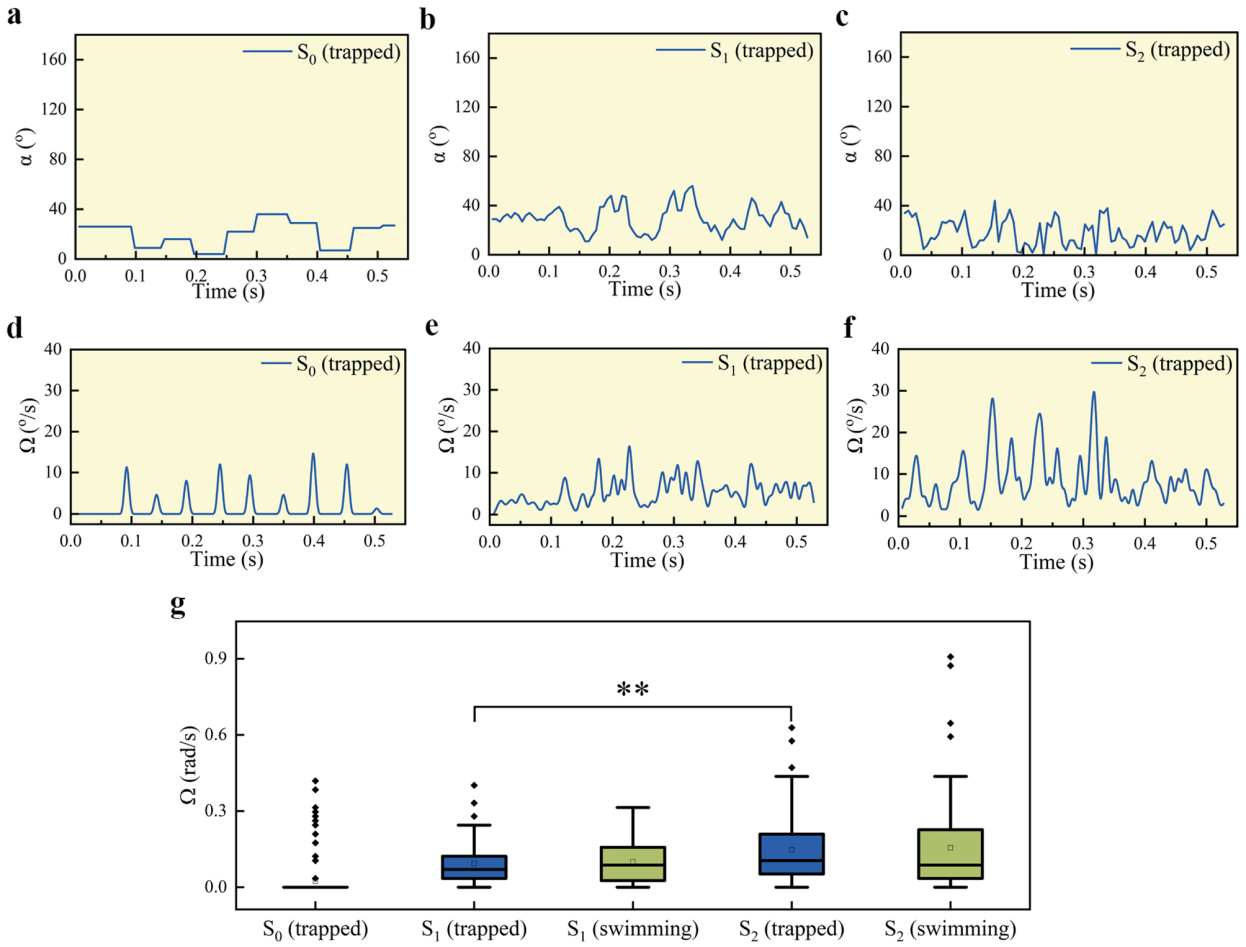
Phase angle signal and corresponding angular speed rate for different motilities of sperm $S_0$ (**a**,**c**), $S_1$ (**b**,**e**), and $S_2$ (**c**,**f**) when they are trapped in the OT. Comparison of angular speed rate for sperms before (green data) and after (blue data) they are trapped. Mean values with 25–75% of data are depicted. The significance level was set at **p < 0.01.

The aforementioned methodology was employed to determine the phase value of a rotating sperm under optical trapping control using sperm samples obtained from a single healthy male. The experiment was conducted at a temperature of 23 $^{\circ }$C. The laser output power was set to 400 mW. The laser trapping power reaching the sperm can be measured with a power meter and is approximately 120 mW, resulting in a temperature increase of around 1 $^{\circ }$C^27–29^. To try to minimize the potential for light-induced damage to the sperm, the capture duration of the trapped sperm was limited to no more than 4 s^30^. To facilitate comparative analysis, we opted to select three representative sperms for evaluation, namely $S_0$, $S_1$, and $S_2$. $S_0$ appeared to be inactive under microscopic observation with minimal or no detectable movement, while both $S_1$ and $S_2$ exhibited rapid head oscillatory movements in a trapped state contrasting with the dead-like state of $S_0$. Figure 3a–c,d–f illustrate the phase information and calculated angular speed rate for the three sperm cells mentioned above . It can be seen that for the basically inactive sperm cell, the signal in Fig. 3d shows low-frequency sparsity, while the signal in Fig. 3e,f is high-frequency density. For the angular speed signal, we used a box plot for statistical analysis. The results are shown in Fig. 3g. It can be seen that for $S_0$, both the mean and median tend to be 0, while for the other two trapped more active sperm cells, their mean and median are relatively larger. Furthermore, we used a two-sample t-test in statistics to determine whether there is a significant difference in the mean angular speed between the two groups. And the results suggest that the rotation activity levels of $S_2$ sperm were likely higher than those of $S_1$ sperm, as indicated by a statistically significant p value of less than 0.01. Accordingly, we could select $S_2$ sperm as the target of our research due to its relatively higher motility levels when compared to $S_1$ sperm.

In addition, we manually tracked the trajectory of the untrapped sperm (about 40 frames) with the aid of ImageJ^24^ and used the method of tracking ROI to provide the angular speed of the swimming sperm prior to capture, as shown in Fig. 3f. It can be seen that the speed distribution of the untrapped sperm signal is larger. This is because the sperm will experience the torque of the optical trap during rotation, which will cause the rotation speed to decrease, as shown in the wider range of 25–75% of the swimming group compared to the trapped group. However, the average output power of trapped and untrapped sperm is basically the same.

**Figure 4:**
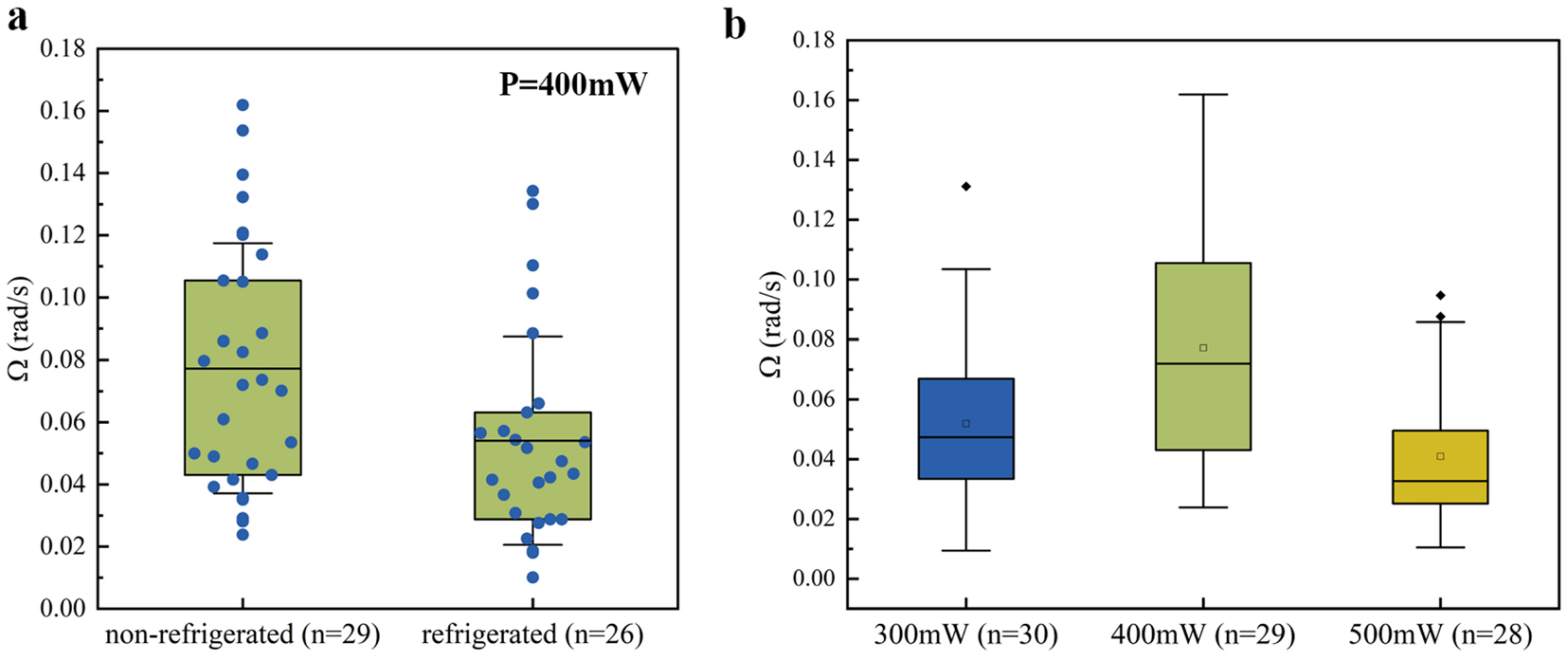
Comparison of sperm angular speed at different temperatures (**a**), with non-refrigerated sperms on the left and refrigerated sperms on the right. Mean values ± SD with 25–75% data (green data and blue dots) are depicted. Comparison of sperm angular speed for different laser output power (**b**). Mean values ± SD with 25–75% data are depicted. In both of them, n represents the total sample number.

In order to further confirm the feasibility of the method. We divided the sperm from the same sample into two parts (about 800 $\upmu$L each), one was refrigerated at 4 $^{\circ }$C for 6 h, and the other was stored at room temperature(23 $^{\circ }$C) for 6 h for statistical analysis of the average angular speed at a laser output power of 400 mW. In order to avoid the increase in motility of the refrigerated sperm caused by rewarming as much as possible, we replaced the sample and captured sperms after 5 captures. The results are shown in Fig. 4a. It can be seen that the average activity of refrigerated sperm is reduced by about 30%, which is consistent with the expected decrease in sperm activity after refrigeration. Meanwhile, we chose 300 mW, 400 mW, and 500 mW as the laser output power with other conditions held constant to acquire the angular speed of trapped sperm under different trapping energy potentials. Regarding the temperature increase caused by optical trapping, due to a difference of 30 mW between actual laser trapping powers, the maximum temperature difference induced by different laser powers remains within 1 $^{\circ }$C^27–29^. This temperature difference has a minimal impact on the experiment. As shown in Fig. 4b, it can be seen that at these power levels, the sperm can be stably trapped. It is evident that at 400 mW, the trapped sperm have the highest average angular speed and widest distribution range. While at 500 mW and 300 mW, their speeds are relatively low and their ranges narrow. This is probably due to two reasons: the torque exerted on the captured target exhibits a positive correlation with the increase in intensity, which is directly linked to the laser output power^31,32^. At 500 mW, the resistance torque is too high, resulting in excessive damping of the sperm and limiting its rotation; while at 300 mW, the energy is too low to capture highly active sperm^33^, resulting in lower captured sperm rotation motility. Therefore, 400 mW is the ideal output power with the largest range of captured sperm rotation motility, and the selected power in practical applications needs to be above the appropriate output power to achieve maximum distinguishability.

## Conclusion

This research paper proposes a deep learning-based method for image segmentation of sperm heads, which enables the dynamic extraction of rotation angular speed as a criterion for optically trapped sperm motility detection. The study demonstrates the reliability of the method by comparing refrigerated and normal sperm samples and identifies the optimal laser power range for maximizing the distinction between sperm. This research provides a strong foundation for subsequent sperm selection using optical tweezers and has a potential for clinical applications such as ICSI, which could improve outcomes for couples facing fertility challenges (Supplementary Information).

### Sample preparation

 The human semen samples were collected from individual patients at the Reproductive Medicine Center, the First Affiliated Hospital of Anhui Medical University. All patients gave informed consent prior to being included in the study. The semen samples were diluted in fertilization medium (Cook Medical, U.S.) and stored at 4 $^{\circ }$C for subsequent experiments. Before loading onto the self-built optical tweezers instrument’s sample pool, the sperm samples were further diluted in 20–40 folds with fertilization medium.

### Statistical analysis

 All analyses were performed using Origin 2021b. A two-sided P value of $<0.05$ was considered significant.

### Ethical approval

 The studies involving human participants were reviewed and approved by Biomedical Ethics Committee of Anhui Medical University. The patients/participants provided their written informed consent to participate in this study. All methods were carried out in accordance with the university guidelines and regulations for sperm experimentation.

### Supplementary Information

Supplementary Legends.

Supplementary Video 1.

Supplementary Video 2.

Supplementary Video 3.

Supplementary Video 4.

## Data Availability

The datasets used and analysed during the current study available from the corresponding author on reasonable request.
