# Supplementary material for: Deep learning-based method for analyzing the optically trapped sperm rotation
Source: Sci Rep. 2023 Aug 3;13:12575. doi: 10.1038/s41598-023-39819-7 (PMC10400645; doi:10.1038/s41598-023-39819-7)
Supplement: Supplementary file 1 — Supplementary Legends. [file 41598_2023_39819_MOESM1_ESM.docx]

**Supplemental visualization 1.** Video of a trapped sperm, which refers to sperm S2, swimming in the ROI(red bounding box).

**Supplemental visualization 2.** Video of sperm motion within ROI from Supplemental Visualization 1.

**Supplemental visualization 3**. Video of the segmented result of Supplemental Visualization 2 by means of U-Net++.

**Supplemental visualization 4**. Video of the elliptical fitting result of Supplemental Visualization 3.
